# Conservative treatments for women with stress urinary incontinence: a systematic review and network meta-analysis

**DOI:** 10.3389/fmed.2024.1517962

**Published:** 2024-12-04

**Authors:** Mengzhen Li, Kexin Qiu, Haoran Guo, Mengsi Fan, Li Yan

**Affiliations:** ^1^School of Public Health, Shandong Second Medical University, Weifang, China; ^2^Department of Gynecology, Shandong Provincial Qianfoshan Hospital, Shandong Second Medical University, Key Laboratory of Laparoscopic Technology, The First Affiliated Hospital of Shandong First Medical University, Jinan, China; ^3^Department of Gynecology, The First Affiliated Hospital of Shandong First Medical University and Shandong Provincial Qianfoshan Hospital, Jinan, China

**Keywords:** stress urinary incontinence, conservative treatment, network meta-analysis, biofeedback electrical stimulation, laser

## Abstract

**Objective:**

This study aimed to compare the effectiveness of various conservative treatment strategies for women with stress urinary incontinence.

**Methods:**

A comprehensive search of PubMed, Web of Science, Embase, and the Cochrane Library was conducted from their inception through March 2024, without restrictions on language or location. Randomized controlled trials (RCTs) comparing the efficacy of conservative treatments for stress urinary incontinence, using short-term pad test or the International Consultation on Incontinence Questionnaire-Urinary Incontinence Short Form (ICIQ-UI SF) score as outcome measures, were included. We conducted a network meta-analysis using a random-effects model to compare the effectiveness of different conservative treatment strategies, employing prediction interval plots and league tables, and ranked them according to the surface under the cumulative ranking curve (SUCRA). The quality of the included studies was assessed following the Cochrane Handbook for Systematic Reviews of Interventions.

**Results:**

A total of 31 RCTs involving 1,900 patients across 8 intervention categories were included in the analysis. SUCRA rankings indicated that electrical stimulation (SUCRA = 95.9%) was the most effective therapy for improving ICIQ-UI SF scores, followed by biofeedback electrical stimulation (SUCRA = 84.9%), radiofrequency (SUCRA = 77.5%), biofeedback (SUCRA = 57.8%), magnetic stimulation (SUCRA = 45.3%), pelvic floor muscle training (SUCRA = 38.4%), Er: YAG laser (SUCRA = 37.4%), and CO_2_ laser (SUCRA = 7.4%). In terms of reducing urine leakage, the treatments were ranked in descending order as follows: Er: YAG laser (SUCRA = 97.5%), biofeedback electrical stimulation (SUCRA = 83.4%), biofeedback (SUCRA = 67.0%), radiofrequency (SUCRA = 59.5%), electrical stimulation (SUCRA = 48.4%), pelvic floor muscle training (SUCRA = 43.0%), magnetic stimulation (SUCRA = 27.8%), and CO_2_ laser (SUCRA = 21.4%). Based on the clustered rankings of the two metrics, biofeedback electrical stimulation was identified as the most effective therapy for improving stress urinary incontinence.

**Conclusion:**

Based on the combined analysis of two indicators, we found that biofeedback electrical stimulation may be the optimal therapy for the conservative management of stress urinary incontinence.

**Systematic review registration:**

https://www.crd.york.ac.uk/prospero/, identifier CRD42024569845.

## Introduction

Stress urinary incontinence refers to the involuntary loss of urine during activities that increase intra-abdominal pressure, such as physical exertion, sneezing, or coughing (1). Two primary mechanisms have been proposed to explain stress urinary incontinence: (1) urethral hypermobility caused by weakened supportive tissues around the urethra, and (2) intrinsic sphincter deficiency (ISD), which involves a malfunctioning urethral sphincter that fails to maintain closure under stress (2). These mechanisms are not dichotomous but rather represent a continuum, with many patients having features of both (3). A US study reported that the prevalence of stress urinary incontinence ranges from approximately 25 to 55%, with the incidence increasing with age. Notably, more than 50% of women aged 60 years and older are affected by this condition (4). Stress urinary incontinence can significantly impair a patient’s quality of life (QOL), contributing to heightened psychological distress, reduced self-esteem, and compromised sexual health (5, 6).

The treatment of stress urinary incontinence includes both surgical and conservative approaches. While surgical treatment is effective, it is associated with a range of potential complications (7). Consequently, conservative treatment has become the first-line option for managing stress urinary incontinence (8). Among conservative treatments, pelvic floor muscle training is recommended as the primary approach. Additionally, other physical therapies, including laser therapy, radiofrequency therapy, magnetic stimulation, electrical stimulation, and biofeedback, are also considered (9). Several studies have previously evaluated the effectiveness of various conservative treatment strategies. However, the available evidence remains limited, and there is considerable uncertainty regarding the efficacy of commonly used conservative treatment methods (10, 11).

Therefore, the aim of this systematic review and network meta-analysis is to compare the short-term efficacy of various conservative interventions for the treatment of stress urinary incontinence.

## Materials and methods

### Protocol and registration

This study was conducted and reported according to the Preferred Reporting Items for Systematic Reviews and Meta-analyses (PRISMA-NMA) statement (12). We registered the systematic review and network meta-analysis in the International Prospective Register of Systematic Reviews, a prospective international registry of systematic reviews under identifying number CRD42024569845. Since all analyses were founded on previously published research, neither ethical review nor patient permission are necessary.

### Data sources and search strategy

Electronic databases including PubMed, Embase, Web of Science, and Cochrane Library were searched with the use of the following keywords and Medical Subject Heading (MeSH) terms: “urinary incontinence, stress,” “pulsed radiofrequency treatment,” “laser therapy,” “magnetic field therapy,” “electric stimulation,” “biofeedback,” and “pelvic floor muscle therapy.” The search will cover the period from the inception of each database through March 2024, with no restrictions on location or language.

### Inclusion and exclusion criteria

RCTs that met all of the following criteria were included: (1) Population: Studies involving women with stress urinary incontinence or mixed incontinence where stress urinary incontinence was the dominant factor. (2) Intervention: Conservative treatments, including one of the following can be included: laser therapy, radiofrequency therapy, magnetic stimulation, electrical stimulation, biofeedback, biofeedback electrical stimulation, or pelvic floor muscle training. (3) Comparison: Studies comparing these interventions to no treatment, placebo, sham, or other inactive control treatments. (4) Outcome: Studies that reported at least one of the following outcomes: short-term pad test results or the International Consultation of Incontinence Questionnaire-Urinary Incontinence Short Form (ICIQ-UI SF) score. Exclusion criteria were as follows: (1) Non-original Studies, such as systematic reviews, narrative reviews, letters, editorials, conference abstracts, and animal experiments. (2) Studies that were inconsistent with the research objectives or were not RCTs. (3) Studies that failed to report the required outcome measures or did not comply with the standard data reporting format. (4) Studies where the full text was unavailable.

### Screening and data extraction

We managed the literature screening process using EndNote X9. After removing duplicates, two researchers independently reviewed the titles and abstracts under mutual blinding. The initial screening was conducted according to the inclusion and exclusion criteria, eliminating studies that did not meet the inclusion criteria. Articles that met the criteria were then reviewed in full text, with cross-checking. Any disagreements were resolved through discussion, exchanging opinions, or consulting a third party. In the case of several publications using the same data set, we included the study with the most complete data and the most extended follow-up.

Data extraction was performed using a standardized form to capture key details from the included studies, including the first author, publication year, patient characteristics, sample size, treatment modality, outcome measures, follow-up period, and critical factors for assessing risk of bias. To ensure data accuracy, two investigators independently conducted the extraction. Any discrepancies were resolved through discussion and, if necessary, by consulting a third investigator to reach consensus.

### Assessment of risk of bias

The risk of bias in each of the included studies was evaluated using the criteria outlined in the Cochrane Handbook for Systematic Reviews of Interventions (13). Seven domains were critically investigated in each included trial, as follows: (1) random sequence generation; (2) allocation concealment; (3) blinding of participants and personnel; (4) blinding of outcome assessment; (5) incomplete outcome data; (6) selective reporting, and (7) other bias. The review of the authors’ judgments was categorized as “low risk,” “high risk,” or “unclear risk” of bias. The assessment process was conducted independently by two assessors, and any disagreements about the quality of the study were resolved by consensus or by a third author.

### Data synthesis and statistical analysis

All the data analyses and the graphical renderings were performed using STATA version 16 and Review Manager 5.3. This systematic review compared eight different conservative treatment modalities (Er: YAG laser, CO_2_ laser, radiofrequency, magnetic stimulation, electrical stimulation, biofeedback, biofeedback electrical stimulation and pelvic floor muscle training). The primary outcome was the ICIQ-UI SF score (subjective outcome), and the secondary outcome was the short-term pad test (objective outcome). The network meta-analysis was conducted using a frequentist approach, as described by White et al. (14). For each outcome of interest, the command < network meta inconsistency > was used to statistically confirm the overall consistency assumption among the networks. Subsequently, local inconsistency tests were performed using the node-splitting method and loop inconsistency detection. When inconsistency was found to be absent in both global and local tests, the consistency assumption was accepted. In this case, the consistency model suggested that the direct and indirect comparisons guaranteed significant results, and the differences between the results were related only to the effects of the intervention and random errors.

When extracted data were reported as median with interquartile range or range, the mean and SD were estimated using the methods described by Luo et al. (15) and Wan et al. (16). For studies that reported data as mean and SD or 95% CI, we applied the technique described by Borenstein et al. (17) to estimate the mean and SD. All data were ultimately expressed as the difference between baseline and endpoint by permutation (17). Pooled metrics were reported as mean difference (MD) or standardized mean difference (SMD) for continuous variables, along with 95% CI, using the random-effects model of Der Simonian and Laird.

We compared the efficacy of the conservative treatments included in this analysis using either the placebo group or the no-treatment group as the reference. First, a network diagram of the evidence was created for each indicator. The evidence network plot displays the number of patients for each intervention through the size of the dots, while the thickness of the lines between interventions represents the number of studies included. A prediction interval plot and league tables were then constructed for each evaluated outcome to compare the effectiveness of different conservative treatment strategies and to rank the treatments to define superiority by means of a ranking plot (Surface Under the Cumulative Ranking curve Area [SUCRA]). Finally, a cluster analysis based on SUCRA values for both outcomes was conducted to generate a cluster ranking map (18). We analyzed potential publication bias by evaluating the symmetry of a “comparison-adjusted” funnel plot.

## Results

### Study selection

**FIGURE 1:**
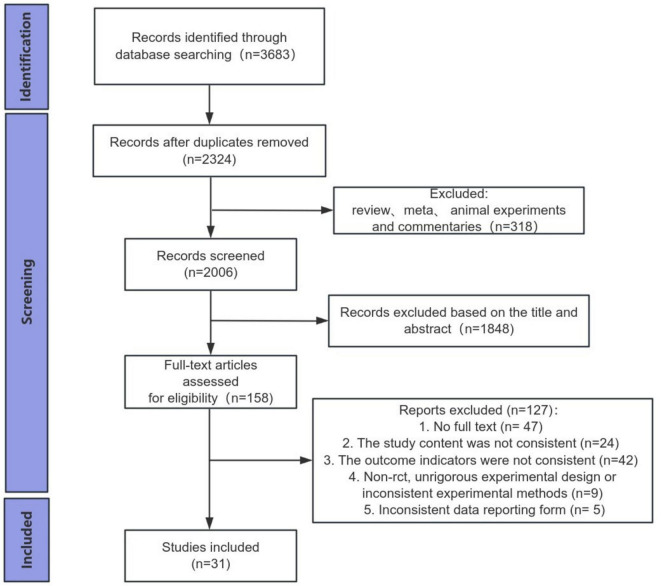
Flow diagram of the study selection process.

3,683 studies were originally identified through database searches. Of these, 1,359 duplicates were removed, and 318 studies were excluded due to being literature reviews, meta-analyses, animal experiments, or commentaries. After title and abstract screening, 1,848 studies were subsequently removed. Full texts of 158 studies were assessed, resulting in the exclusion of 47 studies due to lack of full text, 24 for non-compliance with study content, 42 for not meeting the outcome indicators, 9 for being non-randomized controlled trials, poor trial design, or methodological incompatibility, and 5 for not adhering to the required data reporting format. 31 studies, including 1,900 participants, were included in the quantitative synthesis and network meta-analysis (19–49) (Figure 1).

### Study characteristics

The studies were conducted between 1995 and 2023. All included studies focused on women with stress incontinence or mixed incontinence, with stress incontinence as the dominant factor. Among the 31 studies, 2 (6.45%) utilized Er: laser therapy,(32, 33) 3 (9.68%) employed CO_2_ laser therapy,(29–31) 3 (9.68%) used radiofrequency therapy,(19–21) 7 (22.58%) used magnetic stimulation therapy,(22–28) 7 (22.58%) implemented electrical stimulation therapy,(34–40) 5 (16.13%) used biofeedback therapy,(41–45) and 4 (12.90%) employed biofeedback electrical stimulation therapy (46–49). The duration of treatment varied, with the shortest course being a single session and the longest extending to 24 weeks. Follow-up periods ranged from 1 week to 6 months. Among the 31 studies with outcome measures, 12 (38.71%) reported the ICIQ-UI SF score, 16 (51.61%) utilized the pad test, and 3 (9.68%) included both the ICIQ-UI SF score and the pad test (Table 1).

**TABLE 1:** Characteristics of included studies.

References	Inclusion criteria	Population		Age (mean ± SD)		Intervention		Follow-up	Outcome
		**T**	**C**	**T**	**C**	**T**	**C**		
Leibaschoff et al. (19)	Postmenopausal women with stress urinary incontinence	10	10	55 ± 5.8	56.9 ± 3.1	RF*	Placebo	3 months	ICIQ-UI SF
Slongo et al. (21)	Women aged between 45 and 65 years, with complaints of or mixed urinary incontinence with stress Predominance	36	26	55.33 ± 6.23	55.69 ± 6.14	RF*	PFMT*	1 months	1-h pad-test
									ICIQ-UI SF
Chinthakanan et al. (20)	Postmenopausal women with mild to moderate degree of stress urinary incontinence	23	26	62.96 ± 5.60	59.65 ± 6.31	RF*	Placebo	3 months	1-h pad-test
Lim et al. (24)	Stress urinary incontinence	57	58	51.8 ± 10.0	52.7 ± 7.8	MS*	Placebo	2 months	ICIQ-UI SF
Dudonienë et al. (23)	Women aged between 29 and 49 years, with complaints of stress urinary incontinence	24	24	40.25 ± 6.49	37.58 ± 5.86	MS*	PFMT*	1.5 months	ICIQ-UI SF
Manganotti et al. (25)	Stress urinary incontinence	10	10	50.1 ± 2.86		MS*	Placebo	1 week	1-h pad-test
Yamanishi et al. (28)	Stress urinary incontinence	18	12	NR		MS*	Placebo	2.5 months	ICIQ-UI SF
González-Isaza et al. (26)	Stress urinary incontinence	22	25	53.63 ± 12.32	54.64 ± 10.81	MS*	Placebo	1 months	ICIQ-UI SF
Mikus et al. (27)	Stress urinary incontinence	46	48	47.45 ± 7.4	49.16 ± 7.6	MS*	PFMT*	2 months	ICIQ-UI SF
Gilling et al. (22)	Stress urinary incontinence	35	35	54	54.8	MS*	Placebo	2 months	20-min pad-test
Alexander et al. (29)	Stress urinary incontinence	42	47	51.5 ± 10.6	54.6 ± 11.2	CO_2_ Laser	Placebo	3 months	ICIQ-UI SF
Temtanakitpaisan et al. (31)	Women with stress urinary incontinence and mixed urinary incontinence, stress predominant	28	28	49.7 ± 10.9	52.8 ± 11.8	CO_2_ Laser	Placebo	3 months	ICIQ-UI SF
Lauterbach et al. (30)	Women aged 40–70 years who experienced temporary significant improvement in symptoms following CO_2_ laser treatments for stress urinary incontinence	63	68	51.8 ± 3.5	52.3 ± 3.9	CO_2_ Laser	Placebo	6 months	1-h pad-test
									ICIQ-UI SF
Blaganje et al. (32)	Premenopausal (age range 35–65 years), sexually active women with at least one vaginal delivery and a diagnosis of stress urinary incontinence	56	56	39.95 ± 6.36	41.84 ± 5.67	Er: YAG laser	Placebo	3 months	ICIQ-UI SF
da Fonseca et al. (33)	Postmenopausal women with mild-to-moderate stress urinary incontinence	16	16	57.9 ± 6.1	62.7 ± 9.1	Er: YAG laser	PFMT*	6 months	1-h pad-test
Castro et al. (34)	Stress urinary incontinence	27	24	55.2 ± 12.8	52.6 ± 11.2	ES*	No treatment	6 months	PAD Test volume
		26		56.2 ± 12.5		PFMT*			
Demirtürk et al. (38)	Stress urinary incontinence	20	20	52 ± 7	49+7	ES*	BF*	1.25 months	1-h pad-test
Correia et al. (38)	Women over the age of 60 years, with at least one episode of stress urine leakage during the previous month	7	7	68.57 ± 10.93	69.28 ± 6.94	SES*	No treatment	1.5 months	1-h pad-test
Correia et al. (39)	Women over the age of 50 years, with stress urinary incontinence	15	15	64.46 ± 8.83	60.13 ± 9.35	SES*	No treatment	1.5 months	1-h pad-test
		15		59.86 ± 4.82		IVES*			
Wang et al. (40)	Women with mild-to-moderate stress urinary incontinence	27	30	38.3 ± 4.6	37.7 ± 4.5	ES*	PFMT*	2 months	ICIQ-UI SF
Sand et al. (37)	Genuine stress incontinence (GSI)	28	16	50.9 ± 9.8	57.7 ± 13.3	ES*	Placebo	3 months	20-min pad test
Hwang et al. (36)	Stress urinary incontinence	16	16	42.3 ± 9.1	41.1 ± 7.2	SES*	No treatment	2 months	ultra-short perineal pad test
Mørkved et al. (43)	Stress urinary incontinence	36	34	47.8 ± 8.2	45.4 ± 8.1	BF*	PFMT*	6 months	Standardized pad test
Hirakawa et al. (42)	Stress urinary incontinence	19	20	55.3 ± 9.8	58.3 ± 11.2	BF*	PFMT*	3 months	1-h pad-test
									ICIQ-UI SF
Bertotto et al. (41)	Postmenopausal women with stress urinary Incontinence	16	14	58.4 ± 6.8	57.1 ± 5.3	BF*	No treatment	1 months	ICIQ-UI SF
		15		59.3 ± 4.9		PFMT*			
Ozlu et al. (44)	Mild and moderate severity of stress urinary incontinence	17	17	42.33 ± 9.66	42.82 ± 6.30	Intravaginal BF*	PFMT*	2 months	1-h pad-test
		17		42.11 ± 8.33		perineal BF*			
Fitz et al. (45)	The patients with SUI and/or mixed urinary incontinence with predominant stress urinary incontinence symptoms	30	30	56.1 ± 10.5	56.6 ± 12.0	BF*	PFMT*	3 months	20-min pad test
Kolodynska et al. (48)	Postmenopausal women with stress urinary incontinence	20	20	57.33 ± 6.26		BF*	No treatment	2 weeks	1-h pad-test
Zhu et al. (49)	Stress urinary incontinence	55	55	28.4 ± 3.69	27.66 ± 3.5	BES*	PFMT*	3 months	ICIQ-UI SF
Huebner et al. (46)	Stress urinary incontinence	33	27	49.8 ± 12.9		BF* + conventional ES*	BF*	3 months	Pad weight test
		28				BF* + dynamic ES*			
Terlikowski et al. (47)	Stress urinary incontinence	64	29	46.9 ± 6.8	45.6 ± 7.9	BES*	Placebo	2 months	20-min pad test

*RF, radiofrequency; MS, magnetic stimulation; ES, electrical stimulation; SES, surface electrical stimulation; IVES, intravaginal electrical stimulation; BES, biofeedback electrical stimulation; BF, biofeedback; PFMT, pelvic floor muscle training; NR, not reported.

### Risk of bias of included studies

The quality of the methodology used for each trial is shown in Supplementary Figure 1, and a summary of the quality of methodology expressed in percentages across all trials is represented in Supplementary Figure 2. Almost all studies were at low risk of bias in random sequence generation; 17 (54.84%) were at unclear risk of bias and 14 (45.16%) were at low risk of bias in allocation concealment. Blinding for participants and personnel was clearly described in 9 (29.03%) studies, which was not the case in 13 (41.94%) studies, and 9 (29.03%) studies had a high risk of bias. The risk of bias in blinding for outcome assessment was low, unclear and high in 13 (41.94%), 15 (48.39%) and 3 (9.68%) studies, respectively. Only one study was identified as high risk due to incomplete outcome data, while the remaining studies were classified as low risk. All studies were assessed as low risk for selective reporting and other biases.

### Synthesis of results

#### ICIQ-UI SF score

Fifteen studies assessed the severity of stress incontinence using the ICIQ-UI SF score (19, 21, 23, 24, 26–32, 40–42, 49). The conservative treatments evaluated included Er: laser, CO_2_ laser, radiofrequency, magnetic stimulation, electrical stimulation, biofeedback, biofeedback electrical stimulation, and pelvic floor muscle training. Among the included studies, one was a 3-arm study, while the remaining studies were 2-arm studies, covering a total of eight different interventions.

**FIGURE 2:**
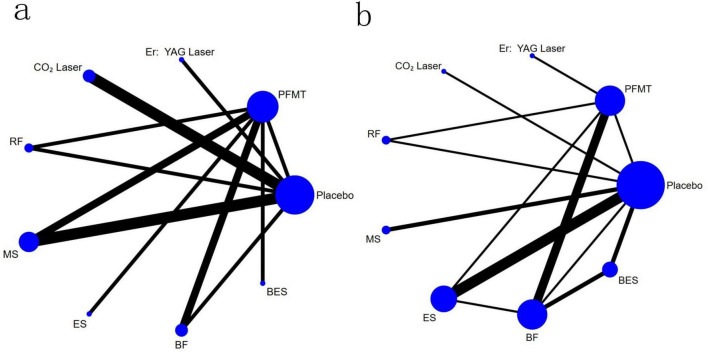
Network plots of comparisons in the network meta-analysis for ICIQ-UI SF scores **(A)** and pad test **(B)** between intervention categories. A relationship map between the interventions was generated based on the data directly compared in the literature. The vertices of the network diagram represent the various interventions in each study, with the size of the vertices corresponding to the sample size of each intervention. The lines in the network diagram indicate direct comparisons between different interventions, with the width of the lines proportional to the number of studies comparing each pair of treatments. RF, radiofrequency; MS, magnetic stimulation; ES, electrical stimulation; BES, biofeedback electrical stimulation; BF, biofeedback; PFMT, pelvic floor muscle training.

The evidence network was centered around no treatment/placebo, as shown in Figure 2A. Three closed loops were identified: placebo–pelvic floor muscle training–magnetic stimulation, placebo–pelvic floor muscle training–radiofrequency, and placebo–pelvic floor muscle training–biofeedback. Among these, magnetic stimulation was the most extensively studied conservative treatment strategy.

Inconsistency analysis showed that global inconsistency was not found (*P* = 0.11). The local inconsistency test, using the node-splitting method, indicated that two direct and indirect estimates differed, potentially due to heterogeneity between studies. However, in the network closure, all *p*-values exceeded 0.05, suggesting good consistency between direct and indirect comparisons within the closed loops. Consequently, we applied the consistency model for the analysis.

For this primary outcome, the “comparison-adjusted” funnel plot (Supplementary Figure 3) showed that all studies were roughly distributed on both sides of the midline, indicating no significant publication bias in this network meta-analysis.

**FIGURE 3:**
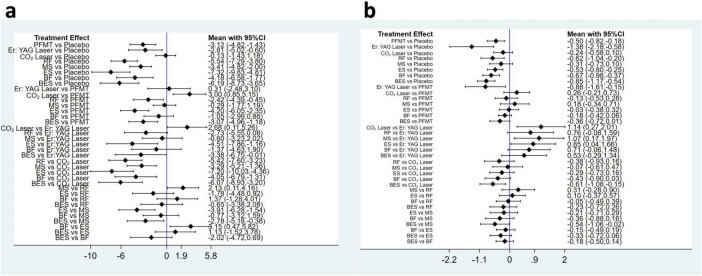
Prediction interval plot of comparisons in the network meta-analysis for ICIQ-UI SF scores **(A)** and pad test **(B)** between intervention categories. RF, radiofrequency; MS, magnetic stimulation; ES, electrical stimulation; BES, biofeedback electrical stimulation; BF, biofeedback; PFMT, pelvic floor muscle training.

The prediction interval plot (Figure 3A) and league table (Table 2) illustrated the effects of various conservative treatment strategies on improving ICIQ-UI SF scores. Electrical stimulation [MD = −3.15, 95% CI (−5.82, −0.47)] was more effective in improving ICIQ-UI SF scores compared to biofeedback. Similarly, when compared to magnetic stimulation, electrical stimulation [MD = −3.91, 95% CI (−6.28, −1.54)], biofeedback electrical stimulation [MD = −2.78, 95% CI (−5.18, −0.38)], and radiofrequency [MD = −2.13, 95% CI (−4.16, −0.11)] demonstrated greater efficacy. Compared to pelvic floor muscle training, electrical stimulation [MD = −4.20, 95% CI (−6.05, −2.35)], biofeedback electrical stimulation [MD = −3.07, 95% CI (−4.96, −1.18)], and radiofrequency [MD = −2.42, 95% CI (−4.39, −0.45)] were more effective. Electrical stimulation [MD = −4.51, 95% CI (−7.86, −1.16)] and biofeedback electrical stimulation [MD = −3.38, 95% CI (−6.75, −0.01)] were also more effective in improving ICIQ-UI SF scores compared to Er: YAG laser therapy. In comparison with CO_2_ laser, interventions including electrical stimulation [MD = −7.20, 95% CI (−10.03, −4.36)], biofeedback electrical stimulation [MD = −6.07, 95% CI (−8.93, −3.20)], radiofrequency [MD = −5.42, 95% CI (−7.60, −3.23)], biofeedback [MD = −4.05, 95% CI (−6.79, −1.31)], magnetic stimulation [MD = −3.29, 95% CI (−5.21, −1.36)], pelvic floor muscle training [MD = −3.00, 95% CI (−5.15, −0.85)], and Er: YAG laser [MD = −2.68, 95% CI (−5.26, −0.11)] were more effective. Compared to placebo, interventions including electrical stimulation [MD = −7.32, 95% CI (−9.83, −4.81)], biofeedback electrical stimulation [MD = −6.19, 95% CI (−8.73, −3.65)], radiofrequency [MD = −5.54, 95% CI (−7.29, −3.80)], biofeedback [MD = −4.18, 95% CI (−6.58, −1.77)], magnetic stimulation [MD = −3.41, 95% CI (−4.82, −2.00)], pelvic floor muscle training [MD = −3.12, 95% CI (−4.82, −1.43)], and Er: YAG laser [MD = −2.81, 95% CI (−5.02, −0.60)] were more effective. Comparisons among other conservative treatment measures did not reveal statistically significant differences.

**TABLE 2:** League table: comparison between conservative interventions for improvement in ICIQ-UI SF scores.

ES+								
−1.13 (−3.78, 1.52)	BES+							
−1.78 (−4.48, 0.92)	−0.65 (−3.38, 2.08)	RF+						
**−3.15** (**−5.82**, **−0.47**)**	−2.02 (−4.72, 0.69)	−1.37 (−4.01, 1.28)	BF+					
**−3.91** (**−6.28**, **−1.54**)**	**−2.78** (**−5.18**, **−0.38**)**	**−2.13** (**−4.16**, **−0.11**)**	−0.77 (−3.12, 1.59)	MS+				
**−4.20** (**−6.05**, **−2.35**)**	**−3.07** (**−4.96**, **−1.18**)**	**−2.42** (**−4.39**, **−0.45**)**	−1.05 (−2.99, 0.88)	−0.29 (−1.77, 1.19)	PFMT+			
**−4.51** (**−7.86**, **−1.16**)**	**−3.38** (**−6.75**, **−0.01**)**	−2.73 (−5.55, 0.08)	−1.37 (−4.63, 1.90)	−0.60 (−3.23, 2.02)	−0.31 (−3.10, 2.48)	Er: YAG Laser		
**−7.20** (**−10.03**, **−4.36**)**	**−6.07** (**−8.93**, **−3.20**)**	**−5.42** (**−7.60**, **−3.23**)**	**−4.05** (**−6.79**, **−1.31**)**	**−3.29** (**−5.21**, **−1.36**)**	**−3.00** (**−5.15**, **−0.85**)**	**−2.68** (**−5.26**, **−0.11**)**	CO_2_ Laser	
**−7.32** (**−9.83**, **−4.81**)**	**−6.19** (**−8.73**, **−3.65**)**	**−5.54** (**−7.29**, **−3.80**)**	**−4.18** (**−6.58**, **−1.77**)**	**−3.41** (**−4.82**, **−2.00**)**	**−3.12** (**−4.82**, **−1.43**)**	**−2.81** (**−5.02**, **−0.60**)**	−0.13 (−1.43, 1.18)	Placebo

*Data are SD (95% CI) of the column treatment relative to the row treatment.

**SD less than 0 favor column treatments. Significant results are in bold. +RF, radiofrequency; MS, magnetic stimulation; ES, electrical stimulation; BES, biofeedback electrical stimulation; BF, biofeedback; PFMT, pelvic floor muscle training.

**FIGURE 4:**
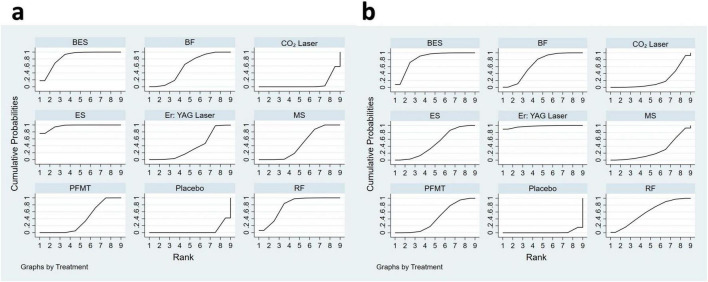
Ranking plot according to SUCRA analysis in the network meta-analysis for ICIQ-UI SF scores **(A)** and pad test **(B)** between intervention categories. The *x*-axis represents the possible rank of each intervention (from the first best rank to the worst according to effectiveness for ICIQ-UI SF scores or pad test). The *y*-axis indicates the cumulative probability for each intervention to be the best intervention, the second-best intervention, the third-best intervention, and so on. In this graphical approach, rankings are presented by examining the area under the curve. The bigger the area under the curve, the higher the likelihood that an intervention is in the top rank or one of the top ranks. RF, radiofrequency; MS, magnetic stimulation; ES, electrical stimulation; BES, biofeedback electrical stimulation; BF, biofeedback; PFMT, pelvic floor muscle training.

Based on the SUCRA analysis for ranking the effectiveness of each intervention in improving ICIQ-UI SF scores, electrical stimulation emerged as the top-ranked therapy (SUCRA = 95.9%), followed by biofeedback electrical stimulation (SUCRA = 84.9%), radiofrequency (SUCRA = 77.5%), biofeedback (SUCRA = 57.8%), magnetic stimulation (SUCRA = 45.3%), pelvic floor muscle training (SUCRA = 38.4%), Er: YAG laser (SUCRA = 37.4%), and CO_2_ laser (SUCRA = 7.4%) (Figure 4A).

### Pad test

Nineteen studies evaluated urine leakage using the pad test (20–22, 25, 30, 33–39, 42–48). The conservative treatments compared in these studies included Er: YAG laser, CO2 laser, radiofrequency, magnetic stimulation, electrical stimulation, biofeedback, biofeedback electrical stimulation, and pelvic floor muscle training. Of these studies, five were three-arm trials, while the remaining were two-arm trials involving eight different interventions. In three of the five 3-arm studies, two groups received the same intervention, so we combined them into a single group (39, 44, 46).

The evidence network, as depicted in Figure 2B, was centered around no treatment/placebo. Six closed loops were identified, including placebo-electrical stimulation-biofeedback treatment, placebo-pelvic floor muscle training-biofeedback treatment, placebo-pelvic floor muscle training-electrical stimulation treatment, placebo-biofeedback-biofeedback electrical stimulation treatment, placebo-pelvic floor muscle training-radiofrequency treatment, and pelvic floor muscle training-electrical stimulation-biofeedback treatment. Among these, biofeedback emerged as one of the most studied conservative treatment strategies.

There was non-significant inconsistency in the overall analysis (*P* = 0.48). In the local inconsistency test, the node-splitting method revealed no significant differences between direct and indirect estimates. The *p*-values within the network’s closed loops were all greater than 0.05, indicating strong agreement between direct and indirect comparisons. Consequently, both the overall and local inconsistency tests were passed, allowing for analysis using the consistency model.

For this secondary outcome, the “comparison-adjusted” funnel plot (Supplementary Figure 4) indicated that all studies were symmetrically distributed around the midline, suggesting a low likelihood of publication bias in this network meta-analysis.

Prediction interval plots (Figure 3B) and league tables (Table 3) were used to compare the effectiveness of various conservative treatment strategies in reducing urine leakage. Er: YAG laser demonstrated greater efficacy in reducing leakage compared to both electrical stimulation [SMD = −0.85, 95% CI (−1.66, −0.04)] and pelvic floor muscle training [SMD = −0.88, 95% CI (−1.61, −0.15)]. Additionally, Er: YAG laser [SMD = −1.07, 95% CI (−1.97, −0.17)] and biofeedback electrical stimulation [SMD = −0.54, 95% CI (−1.06, −0.02)] were more effective than magnetic stimulation. When compared to the CO_2_ laser, both the Er: YAG laser [SMD = −1.14, 95% CI (−2.01, −0.27)] and biofeedback electrical stimulation [SMD = −0.61, 95% CI (−1.08, −0.15)] were more effective. Against placebo, several interventions were more effective: Er: YAG laser [SMD = −1.38, 95% CI (−2.18, −0.58)], biofeedback electrical stimulation [SMD = −0.85, 95% CI (−1.17, −0.54)], biofeedback [SMD = −0.67, 95% CI (−0.98, −0.37)], radiofrequency [SMD = −0.62, 95% CI (−1.04, −0.20)], electrical stimulation [SMD = −0.53, 95% CI (−0.80, −0.25)], and pelvic floor muscle training [SMD = −0.50, 95% CI (−0.82, −0.18)]. No statistically significant differences were observed when comparing the effects of other conservative treatment measures on urine leakage.

**TABLE 3:** League table: comparison between conservative interventions for reducing urine leakage.

Er: YAG Laser								
−0.53 (−1.34, 0.29)	BES+							
−0.71 (−1.48, 0.06)	−0.18 (−0.50, 0.14)	BF+						
−0.76 (−1.59, 0.08)	−0.23 (−0.72, 0.26)	−0.05 (−0.49, 0.39)	RF+					
**−0.85** (**−1.66**, **−0.04**)**	−0.33 (−0.72, 0.06)	−0.15 (−0.49, 0.19)	−0.10 (−0.57, 0.37)	ES+				
**−0.88** (**−1.61**, **−0.15**)**	−0.36 (−0.72, 0.01)	−0.18 (−0.42, 0.06)	−0.13 (−0.53, 0.28)	−0.03 (−0.38, 0.32)	PFMT+			
**−1.07** (**−1.97**, **−0.17**)**	**−0.54** (**−1.06**, **−0.02**)**	−0.36 (−0.88, 0.16)	−0.31 (−0.90, 0.28)	−0.21 (−0.71, 0.29)	−0.18 (−0.71, 0.34)	MS+		
**−1.14** (**−2.01**, **−0.27**)**	**−0.61** (**−1.08**, **−0.15**)**	−0.43 (−0.90, 0.03)	−0.38 (−0.93, 0.16)	−0.29 (−0.73, 0.16)	−0.26 (−0.73, 0.21)	−0.07 (−0.61, 0.47)	CO_2_ Laser	
**−1.38** (**−2.18**, **−0.58**)**	**−0.85** (**−1.17**, **−0.54**)**	**−0.67** (**−0.98**, **−0.37**)**	**−0.62** (−**1.04**, −**0.20**)**	**−0.53** (**−0.80**, **−0.25**)**	**−0.50** (**−0.82**, **−0.18**)**	−0.31 (−0.73, 0.10)	−0.24 (−0.58, 0.10)	Placebo

*Data are SMD (95% CI) of the column treatment relative to the row treatment.

**SMD less than 0 favor column treatments. Significant results are in bold. +RF, radiofrequency; MS, magnetic stimulation; ES, electrical stimulation; BES, biofeedback electrical stimulation; BF, biofeedback; PFMT, pelvic floor muscle training.

The effectiveness of each intervention in reducing urine leakage was ranked using SUCRA analysis. Er: YAG laser (SUCRA = 97.5%) was the highest-ranked therapy, followed by biofeedback electrical stimulation (SUCRA = 83.4%), biofeedback (SUCRA = 67.0%), radiofrequency (SUCRA = 59.5%), electrical stimulation (SUCRA = 48.4%), pelvic floor muscle training (SUCRA = 43.0%), magnetic stimulation (SUCRA = 27.8%), and CO_2_ laser (SUCRA = 21.4%) (Figure 4B).

### Synthesize two outcome indicators clustering ranking

**FIGURE 5:**
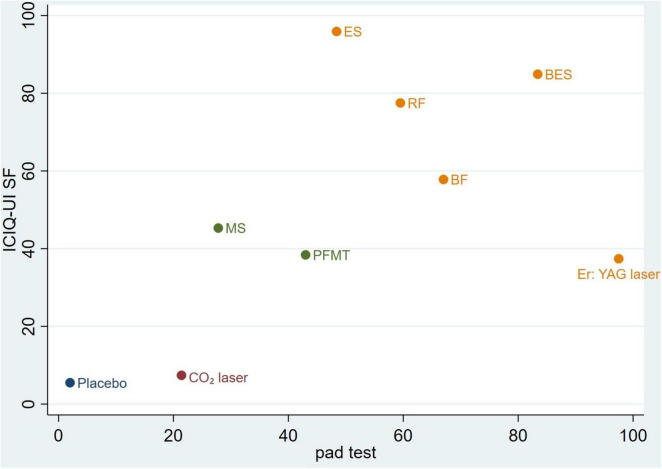
Clustered ranking for the conservative intervention categories on ICIQ-UI SF scores and pad test. The plot is based on the clustered analysis of SUCRA values (horizontal and vertical axes values). Treatments lying in the upper right corner are considered to perform well for both outcomes. Each color represents a group of treatments that belong to the same cluster. RF, radiofrequency; MS, magnetic stimulation; ES, electrical stimulation; BES, biofeedback electrical stimulation; BF, biofeedback; PFMT, pelvic floor muscle training.

Cluster rankings based on changes in ICIQ-UI SF score and pad test leakage indicated that biofeedback electrical stimulation was the most effective therapy for improving symptoms of stress urinary incontinence (Figure 5).

## Discussion

This systematic review and network meta-analysis incorporated 31 randomized controlled trials across eight intervention categories. It evaluated the effectiveness of various conservative treatments in alleviating symptoms and reducing urine leakage in patients with stress urinary incontinence. Er: YAG laser, radiofrequency, magnetic stimulation, electrical stimulation, biofeedback, biofeedback electrical stimulation, and pelvic floor muscle training all significantly reduced leakage and improved symptoms in patients compared to placebo. The combined analysis of these metrics indicated that biofeedback electrical stimulation was the most effective intervention, while CO_2_ laser therapy was the least effective.

Electrical stimulation is employed to improve urinary incontinence by enhancing pelvic floor muscle contractions through both direct stimulation of the pelvic floor muscles and indirect stimulation of the pudendal nerves (50). It includes both intravaginal electrical stimulation and surface electrical stimulation techniques (39). Compared with biofeedback, magnetic stimulation, pelvic floor muscle training, and laser therapy, electrical stimulation was more effective in improving the ICIQ-UI SF score. This suggests that electrical stimulation has a significant impact on relieving patients’ subjective symptoms. This effectiveness may be attributed to electrical stimulation’s role in enhancing bladder and urethral control through neuromodulation. Our study further validated the findings of Moroni et al. (51) and Stewart et al. (52).

According to the SUCRA ranking results, biofeedback electrical stimulation ranked highly in both improving ICIQ-UI SF scores and reducing urine leakage, demonstrating its potential advantages as a conservative treatment for stress urinary incontinence. This may be attributed to its dual mechanism: biofeedback electrical stimulation not only enhances the contractile function of the pelvic floor muscles and the external urethral sphincter through electrical stimulation but also provides feedback that helps patients better understand and manage their pelvic floor muscle activity (50, 53). By combining the benefits of both biofeedback and electrical stimulation, this approach allows patients to optimize training effectiveness through immediate feedback while directly stimulating the muscles, potentially leading to superior efficacy. These results suggest that electrical stimulation and biofeedback electrical stimulation may be more effective in improving urinary incontinence symptoms, particularly in patients with greater severity. This enhanced efficacy may be attributed to electrical stimulation’s superior ability to activate the pelvic floor muscles and improve their contraction capacity, thereby alleviating incontinence symptoms.

The laser treatment consists of two different wavelengths of laser: Erbium (2940 nm) and CO_2_ (10600 nm). Both types of lasers trigger a photo-thermal effect on the vaginal wall. Elevated temperatures denature the highly organized triple helix structure of collagen, and lead to collagen contraction into thicker and shorter fibers and consequently to the induction of neo-collagenesis. This collagen tightening improves thickness, elasticity, and firmness of the vaginal wall, improves the suburethral support, and enhances urinary continence (54). In our study, Er: YAG laser was the top-ranked intervention for reducing urine leakage but ranked lower in improving the ICIQ-UI SF score. This discrepancy may be attributed to several factors. Firstly, differences in inclusion criteria, treatment protocols, and follow-up durations between studies could contribute to varied outcomes (32, 33). Secondly, the short-term pad test may be less sensitive and not fully reflective of patients’ daily activities, while the ICIQ-UI SF questionnaire provides a more comprehensive assessment by considering the impact on daily life, making it more meaningful to patients. Our analysis revealed that CO_2_ laser therapy was the least effective for both outcome measures. Consistent with the study by Zhang et al. (55) we found no significant difference in the efficacy of CO_2_ laser therapy compared to placebo in reducing urine leakage or improving the ICIQ-UI SF score. This suggested that CO_2_ laser therapy may have limited effectiveness in the conservative management of stress urinary incontinence, and clinicians should exercise caution when considering this therapy as a treatment option.

Radiofrequency therapy uses high-frequency alternating current to induce collagen denaturation and regeneration in the deep epithelial layer of the vaginal mucosa, promoting tissue remodeling. This process effectively increases the thickness and elasticity of the connective tissue in the pelvic floor muscles, thereby enhancing muscle strength and improving urinary incontinence symptoms (56, 57). In our study, radiofrequency intervention demonstrated superior efficacy, aligning with findings from other studies (58–60).

Magnetic stimulation is a novel, non-invasive method for stimulating the nervous system. It operates by using a time-varying electric current that flows through a coil, creating a corresponding time-varying magnetic field. This magnetic field induces currents within the patient’s tissues, leading to nerve depolarization and subsequent pelvic floor muscle contractions. Repeated activation of terminal motor nerve fibers and motor endplates enhances muscle strength and endurance. This alteration in pelvic floor muscle group activity effectively increases muscle strength and endurance, contributing to the improvement of urinary incontinence symptoms (61). Our primary outcome, the ICIQ-UI SF score, demonstrated significant efficacy, consistent with findings from previous studies (62–64).

Conservative treatment is not only effective in alleviating symptoms, but it has also become the preferred therapeutic option for many patients due to its non-invasive nature (8). Nevertheless, it is important to know that for elderly patients—particularly those with contraindications to surgery or those who are hesitant to choose long-term exercise regimens—the treatment approach may require appropriate adjustments. For this specific group, vaginal pessary placement can be considered as an alternative treatment. Additionally, for patients who meet the indications and seek rapid improvement in their quality of life, simple obliterative procedures represent a viable option (65). In conclusion, while conservative treatment plays a central role in treating stress urinary incontinence, it is crucial to tailor therapeutic approaches to the individual patient’s needs and specific circumstances.

### Strengths and limitations

A key strength of our study is the quality of the included studies, as only randomized trials with an overall low risk of bias were selected for quantitative analysis. We excluded quasi-randomized trials, open trials, and observational studies. Another strength lies in the comprehensive inclusion of nearly all types of conservative treatments, such as laser therapy, radiofrequency, magnetic stimulation, electrical stimulation, biofeedback, biofeedback electrical stimulation, and pelvic floor muscle training.

This study has several limitations. Firstly, due to the limited number of RCTs, we did not impose restrictions on patient age or severity of incontinence, and subgroup analyses based on stress urinary incontinence severity (e.g., mild, moderate, severe) were not conducted. The included studies varied significantly in terms of treatment regimens, duration, and follow-up periods. Secondly, some interventions were supported by only 2 to 3 studies, which may have affected the reliability of our findings. Thus, further research on stress urinary incontinence is warranted. Thirdly, the majority of included studies had follow-up periods of less than 6 months, limiting our analysis to long-term efficacy. Future research should extend follow-up durations to evaluate long-term outcomes. In particular, additional high-quality RCTs are needed to better define the indications and efficacy of laser therapy for stress urinary incontinence. Moreover, future studies should focus on individualized treatments for different patient populations to address varying treatment needs.

## Conclusion

In this network meta-analysis of eight intervention strategies, combining two study metrics, biofeedback electrical stimulation demonstrated a clear advantage in the conservative treatment of stress urinary incontinence and remained the most established and effective treatment option available. However, clinical decisions should be individualized, considering the specific needs of patients as well as the feasibility and long-term efficacy of the treatment. Future research should focus on addressing the limitations of current studies, and there is a need for large-scale, high-quality randomized controlled trials to confirm the true efficacy of biofeedback electrical stimulation.

## Data Availability

The raw data supporting the conclusions of this article will be made available by the authors, without undue reservation.
