# Supplementary material for: Conservative treatments for women with stress urinary incontinence: a systematic review and network meta-analysis
Source: Front Med (Lausanne). 2024 Dec 4;11:1517962. doi: 10.3389/fmed.2024.1517962 (PMC11656316; doi:10.3389/fmed.2024.1517962)
Supplement: Supplementary file 1 [file Data_Sheet_1.docx]

Supplementary Material

**Supplementary Figure 1. Summary of the risk of bias for every trial**


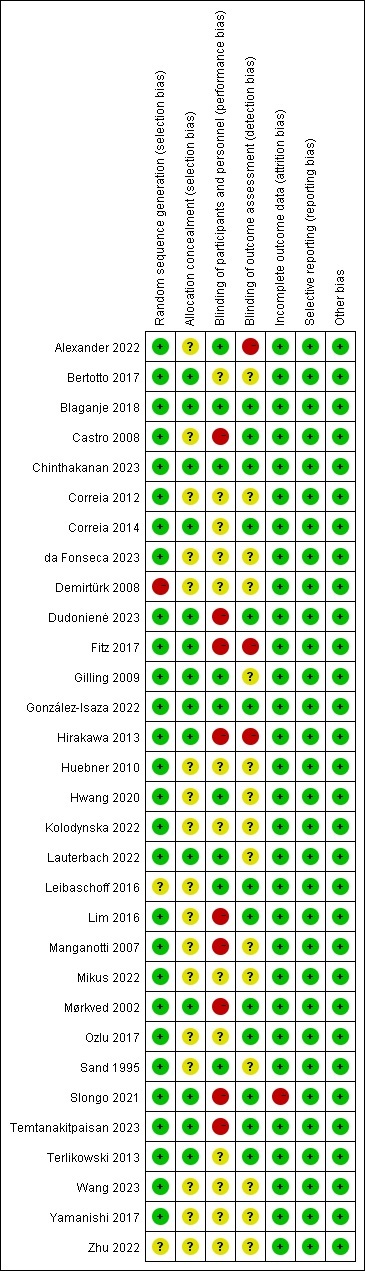


plus sign: low risk of bias; minus sign: high risk of bias;question mark: unclear risk of bias.

**Supplementary Figure 2. Risk of bias graph about each risk of bias item presented as percentages across all included studies.**


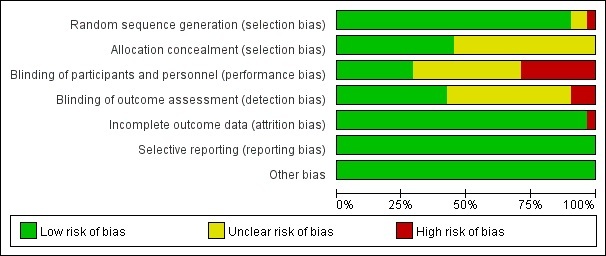


**Supplementary Figure 3.** **Comparison adjusted funnel plot for ICIQ-UI SF scores.**


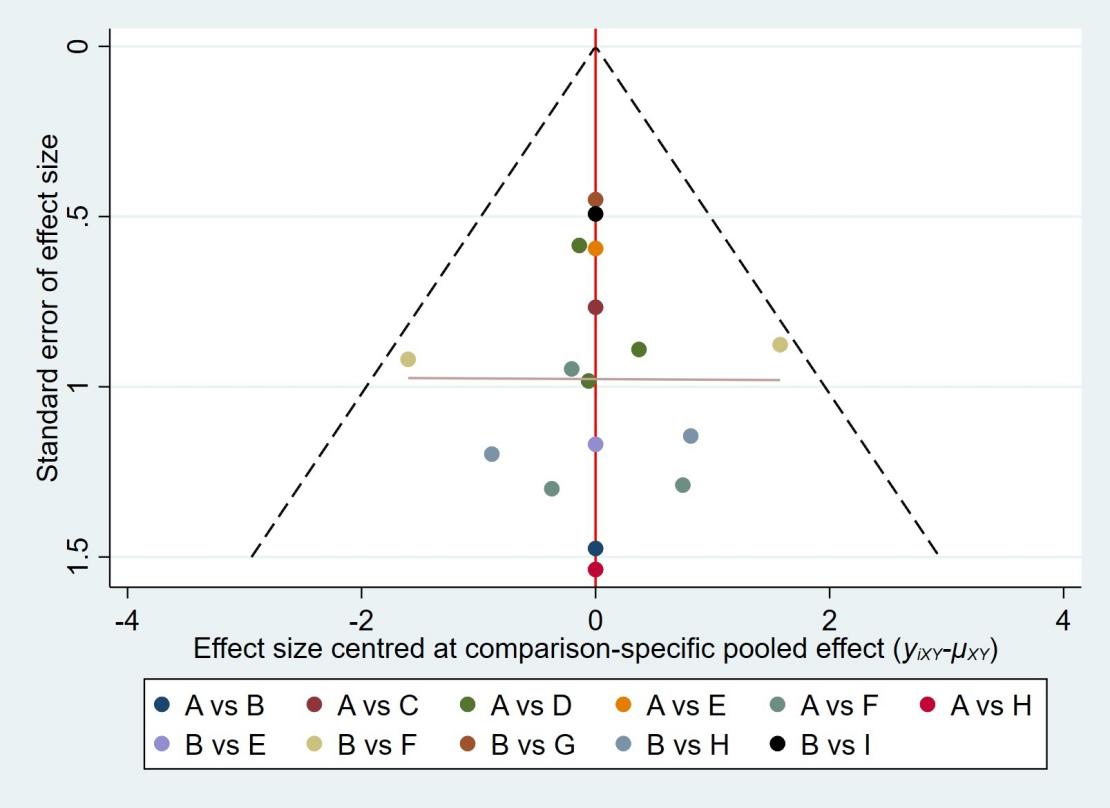


**Supplementary Figure 4 Comparison adjusted funnel plot for pad test.**


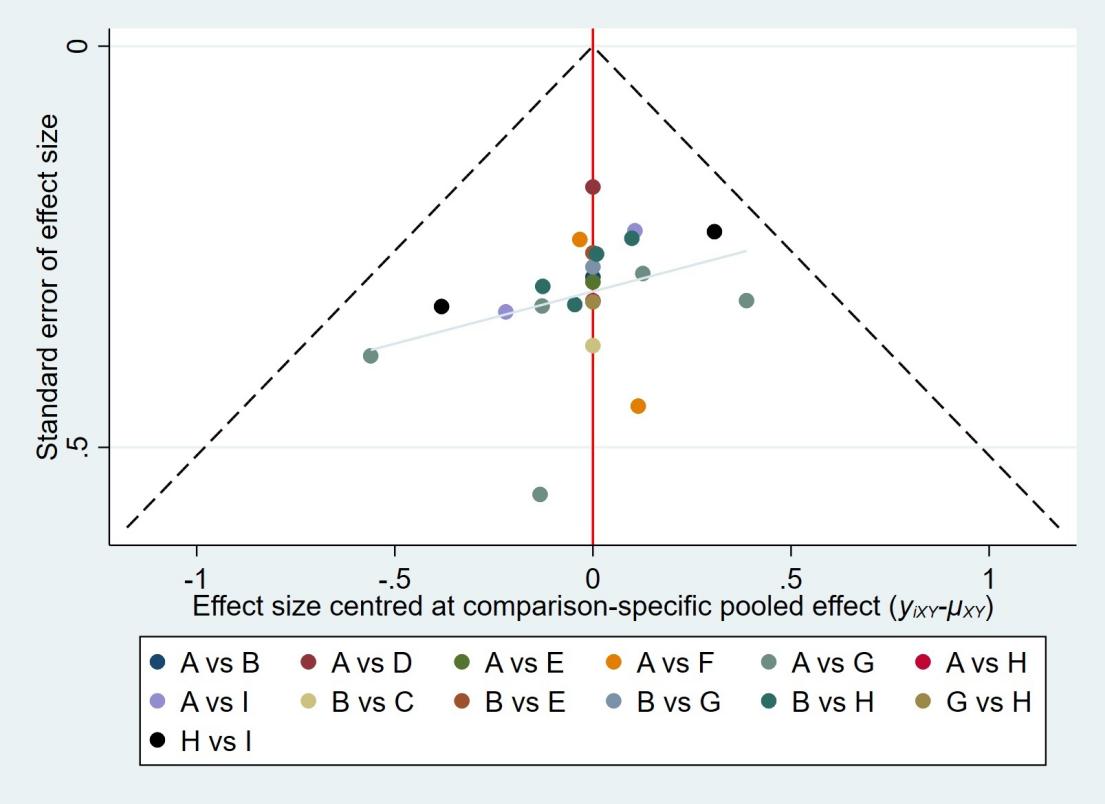


**Supplementary. Pubmed Search strategy**

**Radiofrequency：****searched on March 18,2024.**

(("Urinary Incontinence, Stress"[Mesh]) OR (((Urinary Stress Incontinence[Title/Abstract]) OR (Incontinence, Urinary Stress[Title/Abstract])) OR (Stress Incontinence, Urinary[Title/Abstract]))) AND (((("Pulsed Radiofrequency Treatment"[Mesh]) OR ("Radiofrequency Ablation"[Mesh])) OR ("Radiofrequency Therapy"[Mesh])) OR (((((((((((((((((((Pulsed Radiofrequency Treatments[Title/Abstract]) OR (Radiofrequency Treatment, Pulsed[Title/Abstract])) OR (Radiofrequency Treatments, Pulsed[Title/Abstract])) OR (Treatment, Pulsed Radiofrequency[Title/Abstract])) OR (Treatments, Pulsed Radiofrequency[Title/Abstract])) OR (Pulsed Radio Frequency Treatment[Title/Abstract])) OR (Ablation, Radiofrequency[Title/Abstract])) OR (Radio Frequency Ablation[Title/Abstract])) OR (Ablation, Radio Frequency[Title/Abstract])) OR (Radio-Frequency Ablation[Title/Abstract])) OR (Ablation, Radio-Frequency[Title/Abstract])) OR (Radiofrequency Therapies[Title/Abstract])) OR (Therapies, Radiofrequency[Title/Abstract])) OR (Therapy, Radiofrequency[Title/Abstract])) OR (Radio-Frequency Therapy[Title/Abstract])) OR (Radio Frequency Therapy[Title/Abstract])) OR (Radio-Frequency Therapies[Title/Abstract])) OR (Therapies, Radio-Frequency[Title/Abstract])) OR (Therapy, Radio-Frequency[Title/Abstract])))

**Laser：searched on March 18,2024.**

(("Urinary Incontinence, Stress"[Mesh]) OR (((Urinary Stress Incontinence[Title/Abstract]) OR (Incontinence, Urinary Stress[Title/Abstract])) OR (Stress Incontinence, Urinary[Title/Abstract]))) AND ((("Laser Therapy"[Mesh]) OR ("Lasers"[Mesh])) OR ((((((((((((((((((((((((((((((((((((((((((((((((Laser Therapies[Title/Abstract]) OR (Therapies, Laser[Title/Abstract])) OR (Therapy, Laser[Title/Abstract])) OR (Vaporization, Laser[Title/Abstract])) OR (Laser Vaporization[Title/Abstract])) OR (Laser Ablation[Title/Abstract])) OR (Ablation, Laser[Title/Abstract])) OR (Laser Tissue Ablation[Title/Abstract])) OR (Ablation, Laser Tissue[Title/Abstract])) OR (Tissue Ablation, Laser[Title/Abstract])) OR (Pulsed Laser Tissue Ablation[Title/Abstract])) OR (Laser Photoablation of Tissue[Title/Abstract])) OR (Nonablative Laser Treatment[Title/Abstract])) OR (Laser Treatment, Nonablative[Title/Abstract])) OR (Laser Treatments, Nonablative[Title/Abstract])) OR (Nonablative Laser Treatments[Title/Abstract])) OR (Laser Scalpel[Title/Abstract])) OR (Laser Scalpels[Title/Abstract])) OR (Scalpel, Laser[Title/Abstract])) OR (Scalpels, Laser[Title/Abstract])) OR (Laser Knives[Title/Abstract])) OR (Knive, Laser[Title/Abstract])) OR (Knives, Laser[Title/Abstract])) OR (Laser Knive[Title/Abstract])) OR (Laser Knife[Title/Abstract])) OR (Knife, Laser[Title/Abstract])) OR (Knifes, Laser[Title/Abstract])) OR (Laser Knifes[Title/Abstract])) OR (Laser Surgery[Title/Abstract])) OR (Laser Surgeries[Title/Abstract])) OR (Surgeries, Laser[Title/Abstract])) OR (Surgery, Laser[Title/Abstract])) OR (Laser[Title/Abstract])) OR (Q-Switched Lasers[Title/Abstract])) OR (Laser, Q-Switched[Title/Abstract])) OR (Lasers, Q-Switched[Title/Abstract])) OR (Q Switched Lasers[Title/Abstract])) OR (Q-Switched Laser[Title/Abstract])) OR (Pulsed Lasers[Title/Abstract])) OR (Laser, Pulsed[Title/Abstract])) OR (Lasers, Pulsed[Title/Abstract])) OR (Pulsed Laser[Title/Abstract])) OR (Continuous Wave Lasers[Title/Abstract])) OR (Continuous Wave Laser[Title/Abstract])) OR (Laser, Continuous Wave[Title/Abstract])) OR (Lasers, Continuous Wave[Title/Abstract])) OR (Masers[Title/Abstract])) OR (Maser[Title/Abstract])))

**Magnetic Stimulation:searched on March 18,2024.**

(("Urinary Incontinence, Stress"[Mesh]) OR (((Urinary Stress Incontinence[Title/Abstract]) OR (Incontinence, Urinary Stress[Title/Abstract])) OR (Stress Incontinence, Urinary[Title/Abstract]))) AND ((("Magnetic Field Therapy"[Mesh]) OR (("Magnetic Field Therapy"[Mesh]) OR ((((((((((((((((((((Magnetic Field Therapies[Title/Abstract]) OR (Therapies, Magnetic Field[Title/Abstract])) OR (Therapy, Magnetic Field[Title/Abstract])) OR (Electrically-Charged Magnetic Therapy[Title/Abstract])) OR (Electrically Charged Magnetic Therapy[Title/Abstract])) OR (Electrically-Charged Magnetic Therapies[Title/Abstract])) OR (Magnetic Therapies, Electrically-Charged[Title/Abstract])) OR (Magnetic Therapy, Electrically-Charged[Title/Abstract])) OR (Therapy, Electrically-Charged Magnetic[Title/Abstract])) OR (Magnetic Stimulation Therapy[Title/Abstract])) OR (Magnetic Stimulation Therapies[Title/Abstract])) OR (Stimulation Therapy, Magnetic[Title/Abstract])) OR (Therapies, Magnetic Stimulation[Title/Abstract])) OR (Therapy, Magnetic Stimulation[Title/Abstract])) OR (Magnetotherapy[Title/Abstract])) OR (Static Magnetic Field Therapy[Title/Abstract])) OR (Electromagnetic Therapy[Title/Abstract])) OR (Electromagnetic Therapies[Title/Abstract])) OR (Therapies, Electromagnetic[Title/Abstract])) OR (Therapy, Electromagnetic[Title/Abstract])))) OR (extracorporeal magnetic innervation[Title/Abstract]))

**Electrical Stimulation+Biofeedback+Biofeedback Electrical Stimulation+Pelvic Floor Muscle Training:searched on May 11,2024.**

((("Urinary Incontinence, Stress"[Mesh]) OR ((((stress incontinence[Title/Abstract]) OR (Urinary Stress Incontinence[Title/Abstract])) OR (Incontinence, Urinary Stress[Title/Abstract])) OR (Stress Incontinence, Urinary[Title/Abstract]))) AND (((((((((((Electric Stimulation[Title/Abstract]) OR (electrostimulation[Title/Abstract])) OR (Electrical Stimulation[Title/Abstract])) OR (Electrical Stimulations[Title/Abstract])) OR (Stimulation, Electrical[Title/Abstract])) OR (Stimulations, Electrical[Title/Abstract])) OR (Stimulation, Electric[Title/Abstract])) OR (Electric Stimulations[Title/Abstract])) OR (Stimulations, Electric[Title/Abstract])) OR (((biofeedback[Title/Abstract]) OR (biofeedbacks[Title/Abstract])) OR (EMG[Title/Abstract]))) OR (((((((((((pelvic floor muscle therapy[Title/Abstract]) OR (pelvic floor muscle physiotherapy[Title/Abstract])) OR (pelvic floor muscle exercise[Title/Abstract])) OR (pelvic muscle physiotherapy[Title/Abstract])) OR (pelvic muscle therapy[Title/Abstract])) OR (pelvic muscle exercise[Title/Abstract])) OR (pelvic muscle physical therapy[Title/Abstract])) OR (pelvic floor muscle physical therapy[Title/Abstract])) OR (pelvic floor muscle training[Title/Abstract])) OR (PFMT[Title/Abstract])) OR (pelvic muscle training[Title/Abstract])))) AND ((((((((((randomized controlled trial[Title/Abstract]) OR (randomised controlled trial[Title/Abstract])) OR (randomized[Title/Abstract])) OR (randomised[Title/Abstract])) OR (randomly[Title/Abstract])) OR (controlled[Title/Abstract])) OR (controlled clinical trial[Title/Abstract])) OR (trial[Title/Abstract])) OR (placebo[Title/Abstract])) OR (RCT[Title/Abstract]))
